# Heavy Metal Nanoparticle Detection in Human and Formula Milk

**DOI:** 10.3390/foods13193178

**Published:** 2024-10-06

**Authors:** Antonietta Morena Gatti, Ebe D’Adamo, Valentina Botondi, Stefano Montanari, Erika Colletti, Luigi Gagliardi, Sabina Ciotti, Ali Saber Abdelhameed, Francesca Gazzolo, Antonio Maconi, Rocco Mangifesta, Simonetta Picone, Federico Lauriola, Diego Gazzolo

**Affiliations:** 1 Nanodiagnostics Foundation, 41100 Modena, Italy; gatti@nanodiagnostics.it (A.M.G.); montanari@nanodiagnostics.it (S.M.); 2 Neonatal Intensive Care Unit, G. d’Annunzio University, 65100 Chieti, Italy; ebe.dadamo@yahoo.com (E.D.); valentina.botondi.91@gmail.com (V.B.); erika13colletti@gmail.com (E.C.); federicolauriola@hotmail.it (F.L.); 3 Department of Pediatrics and Neonatology, Versilia Hospital, 55049 Versilia, Italy; luigi.gagliardi@uslnordovest.toscana.it (L.G.); sabina.ciotti@uslnordovest.toscana.it (S.C.); 4 Department of Pharmaceutical Chemistry, College of Pharmacy, King Saud University, P.O. Box 2457, Riyadh 11451, Saudi Arabia; asaber@ksu.edu.sa; 5 Department of Pediatrics, Magna Graecia University, 88100 Catanzaro, Italy; francesca.gazzolo@libero.it; 6 Social Security Administration Development and Promotion of Scientific Research Unit, SS Antonio, Biagio and C. Arrigo Hospital, 15121 Alessandria, Italy; amaconi@ospedale.al.it; 7 Health and Safety Manager Unit, ASL02 Abruzzo, 66100 Chieti, Italy; rocco.mangifesta@asl2abruzzo.it; 8 Neonatology and Neonatal Intensive Care Unit, Policlinico Casilino General Hospital, 00169 Rome, Italy; simpico@libero.it

**Keywords:** breast milk, environmental exposure, formula milk, heavy metal, nanoparticles

## Abstract

Breast milk is the natural source of nutrition for infants, but while it supports their health, it can also be a potential source of toxic inorganic particulate matter, and this applies to both breast milk and industrially produced milk. The aim of the present study was to evaluate the presence of nanoparticles in both breast milk and formula milk samples. We collected and analyzed, via a new electron scanning microscopic procedure, 19 samples of breast milk from Italian women and 19 formula milk samples produced by different companies. Organic–inorganic agglomerates were detected in 58% of formula and in 63% of breast milk samples, respectively. In addition, a significantly (*p* < 0.05) greater size of nanoparticles was observed in formula milk samples. The results, showing the presence of inorganic nanosized particles in breast and artificial milk, may lead to future studies aimed at investigating possible nanosized contamination of milk and identifying early prevention strategies for women and animals involved in the food chain.

## 1. Introduction

In the last decade, inorganic nanoparticle (NP) pollution, particularly but not exclusively involving heavy metals (HM), has emerged as a critical issue as it causes 6.67 million deaths every year and accounts for 12% of all annual deaths globally [1,2].

NPs are officially defined as nano-objects with a size of 1 to 100 nm, commonly classified according to their composition as either organic or inorganic [1]. The latter, due to their ability to interact with human proteins, enzymes, and DNA, has been shown to be a potential health risk in humans from fetal life up to adulthood [3,4] because NPs are easily inhaled or ingested, thus entering the systemic circulation and being stored in different tissues [1,2]. This is particularly noteworthy, especially in the perinatal period, when NPs have been found in cases complicated by early miscarriage, impaired fetal growth, and DNA methylation [5,6]. Results have confirmed that, once NPs are inhaled/ingested, they are carried via maternal systemic circulation and subsequently, through the placenta, enter the fetal bloodstream [7,8,9,10].

In the postnatal period, it has been demonstrated that NPs can enter the body either through inhalation or through food, cosmetics, toothpaste, and drugs to which the mother has been exposed [11,12], supporting the notion that infants may be potentially exposed to NPs through breast milk (BM) and/or industrial infant formulas (FM) obtained from milk of animal origin [13,14,15,16]. In this regard, the Food and Drug Administration (FDA) and the European Medicine Agency (EMA) have issued specific recommendations for the handling of cow’s milk and for its industrial use [17].

Although BM is both unique and the best product for infant nutrition due to its well-known properties as stated by the World Health Organization (WHO), little is known about its possibility of being polluted by NPs [18,19]. This applies to both human and animal milk if whoever produces it lives in a polluted environment or eats polluted foods [20,21].

The detection of inorganic particulate matter in BM and FM samples as described here was performed using a novel environmental scanning electron microscopy technique [22].

## 2. Materials and Methods

### 2.1. Sample Collection

We performed a prospective, multicenter, observational study at our tertiary referral Centers for Obstetrics and Neonatal Intensive Care (NICU) between January 2019 and December 2019. This study was approved by the local ethics committee, and the mothers signed their informed consent (Comitato Etico Interaziendale Presap.ASO.Neonat.19).

We collected BM samples from 19 healthy breastfeeding mothers living in well-known industrial regions of a developed country. None of the mothers included in this study were occupationally exposed to chemicals. We also collected 19 FM samples (preterm formula: n = 10; term formula: n = 9) from different manufacturers, all of whom followed the official institutional guidelines for the handling of cow’s milk.

From each mother, we collected information about the characteristics of the mother herself and the newborn. The main outcomes are reported in Table 1.

All mothers were healthy throughout pregnancy, and no previous abnormal obstetrical history was reported. Appropriate growth was defined by the presence of ultrasonographic markers in accordance with current guidelines and by postnatal confirmation of a birthweight (BW) ranging between the 10th and 90th percentiles according to our Italian population standards after adjusting for the mother’s height, weight, parity, and the sex of the newborn [23,24]. At birth, healthy newborns were defined by the following perinatal outcomes: no maternal illness, no signs of fetal distress, a pH greater than 7.2 in cord or venous blood, and Apgar scores higher than 7 at 1 and 5 min.

The exclusion criteria were maternal infections; tobacco, drug, and alcohol addiction; use of pharmacologically active substances; blood transfusions or use of hematological products; fetal malformations; and chromosomal abnormalities.

### 2.2. NP Chemical–Physical Identification

All samples collected were observed through a scanning electron microscope (ESEM, Quanta 200, Thermofisher, Washington, DC, USA) to identify the morphology of any foreign bodies found. The elemental composition of each inorganic body found was analyzed through the X-ray microprobe of the Energy Dispersive Spectroscope (EDS, EDAX, Mahwah, NJ, USA) coupled to the instrument.

The samples were kept at 4 °C for a week, and in a flow cabinet, a volume of 20 μL was deposited on a 25 mm diameter cellulose filter (Type AA, 0.25 m, Merck, Burlington, MA, USA). The filter was placed on an aluminum stub with an adhesive conductive carbon disk for electron microscopy. All samples were sealed in a clean box, opened only to insert them in the chamber of the ESEM. The investigations were performed in the following conditions: low vacuum 0.2–1.2 Torr, secondary (SE) and backscattered electron mode (BSE), accelerating voltages 12 to 30 kV, wide distance 10 mm, spot 3.8; point-to-point resolution from 10.0 to 4 nm. Debris with a different composition from the organic substrate based on their atomic density are observed as bright point.

Whenever inorganic particles were identified, the X-ray microprobe was used to identify their elemental composition.

### 2.3. Statistical Analysis

Clinical data were expressed as the mean ± SD. A comparison between BM and FM NP concentrations and number was performed by the student’s *t*-test and Mann–Whitney U two-sided test when the data were not normally distributed. A comparison between proportions was performed with the Fisher’s exact test. A *p* < 0.05 was considered significant.

## 3. Results

In Table 1, the perinatal characteristics of the mother–newborn dyad are reported. Maternal age, weight, and height were within reference curves for Caucasian ethnicity. All mothers came from different industrial regions of Italy: six cases from northern, five cases from central, and eight cases from southern regions of Italy. At the time when mature BM samples were collected, all infants were exclusively fed by BM. All mothers followed a balanced Mediterranean diet, and none of them were smokers and/or alcohol consumers.

All infants were healthy and born at term through vaginal delivery. BW was within reference ranges for Italian standards (Table 1). All recruited infants had an Apgar score at 1–5′ ≥ 7; clinical and laboratory standard-of-care parameters were within normality ranges. All infants were discharged from the hospital in normal conditions, and no overt organ damage was detectable.

### 3.1. NP Content Determination in BM

**Figure 1:**
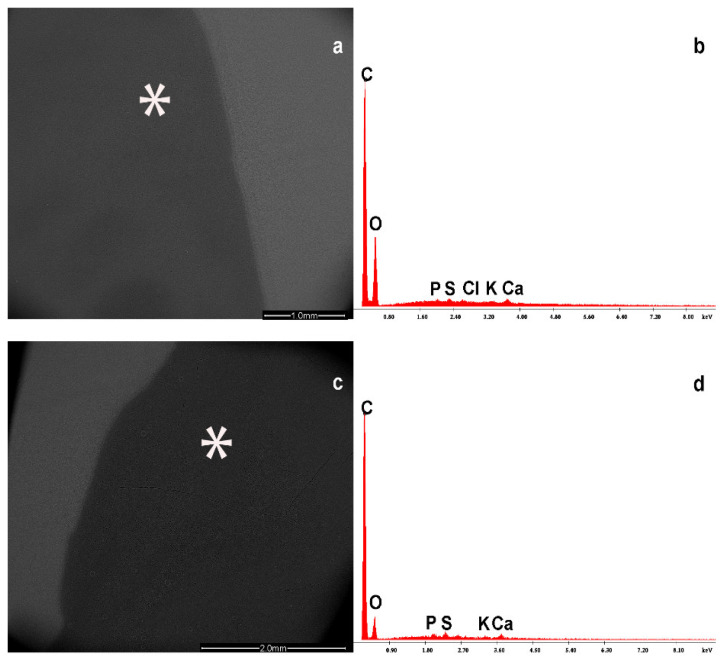
The images (**a**,**c**) show the physiological milk drop and its content (**b**,**d**) in evaporated milk sample taken with an electron microscope on a filter and its EDS spectrum. In some cases, the peak of Chlorine is present. The * symbol shows where the X ray microprobe was localized. Abbreviations: C = carbon; O = oxygen; P = phosphorus; S = sulfur; Cl = chlorine; K = potassium; Ca = calcium.

The physiological BM content is characterized by the presence of elements such as carbon (C), oxygen (O), sulfur (S), calcium (Ca), potassium (K), chlorine (Cl), and phosphorus (P) (Figure 1). Of course, these elements are combined to form protein components, such as casein, vegetable oils, lactose, carbohydrate, vitamins, and oligosaccharides. The EDS system detects only the percentage of the elements and their relative concentration.

Beyond this common substrate, notably, some other organic elements, such as magnesium (Mg) and sodium (Na), were detected. In some samples, the substrate appears grey without any other alteration, while in others, bright spots are detectable. In fact, in 12 cases (63%), a series of white dots (particles) appear with an atomic density higher than the organic substrate. All the EDS spectra contain different amounts of carbon and oxygen that belong also to the common substrate, so we will put attention to the other elements. They are composed of other elements, such as barium (Ba), titanium (Ti), zinc (Zn), rhodium (Rh), palladium (Pd), gold (Au), copper (Cu), nickel (Ni), iron (Fe), chromium (Cr), silicon (Si), aluminum (Al), and manganese (Mn).

The elemental composition of the NPs detected in the BM samples by means of EDS spectrum is reported in Table 2.

**Figure 2:**
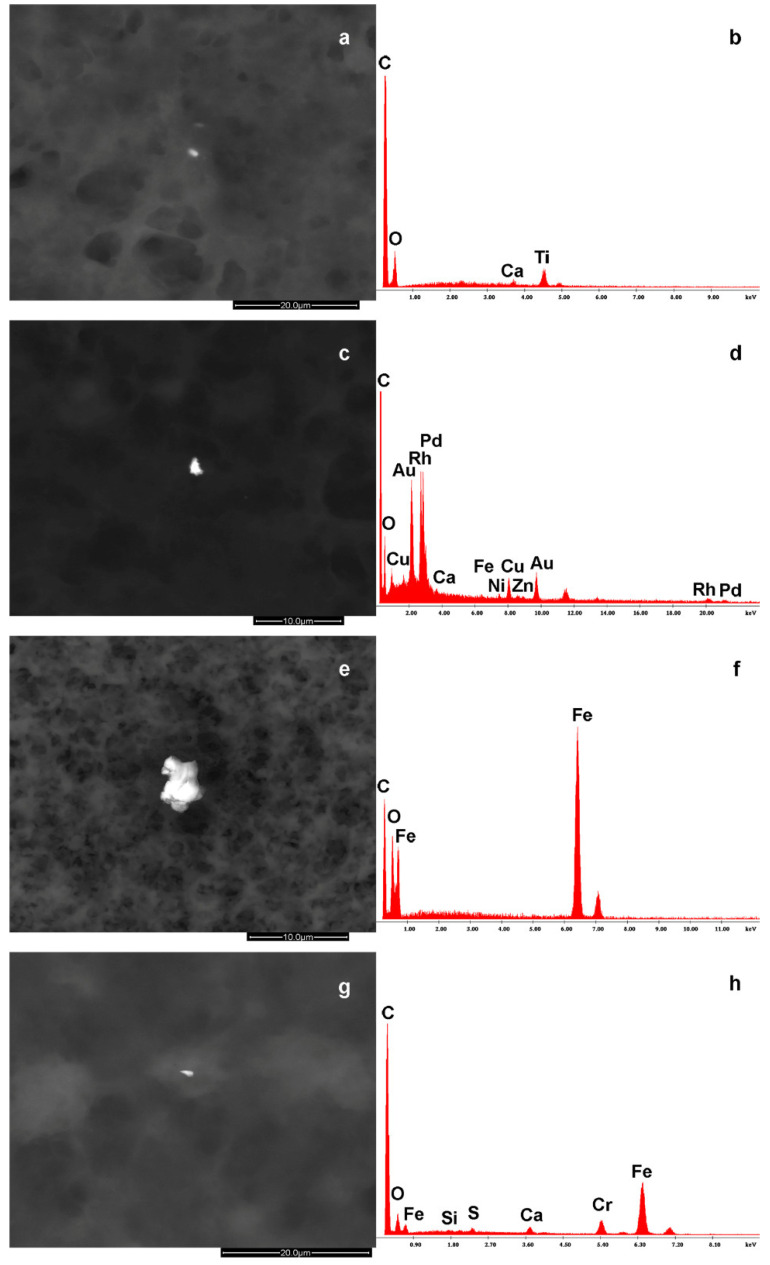
The images show some of the debris found in the breast milk with their EDS spectra. The images (**a**,**b**) represent a carbon (C)–oxygen (O)–calcium (Ca)–titanium (Ti) particle. The images (**c**,**d**) show a C–O–copper (Cu)–gold (Au)–rhodium (Rh)–palladium (Pa)–Ca-iron (Fe)–nickel (Ni)–zinc (Zn)–Ti particle. The images (**e**,**f**) represent a C–O–Fe particle, while the images (**g**,**h**) indicate a C–Fe–chromium (Cr)–Ca–silicon (Si)–sulfur (S) particle that is stainless-steel debris.

In Table 3, the complete composition of the microparticles and NPs identified in the BM samples is reported. The NP size range was below 1 up to 6 μm, regardless of the characteristics of individual elements. Notably, agglomerates resulted from a micro- and nano-biointeraction between organic compounds and inorganic particulate matter. They are foreign bodies and were characterized by the presence of elements such as Zn, Ti, Rh, Pd, Au, Cu, Ni, Fe, S, and Ba (Figure 2, panels A, B, and C).

### 3.2. NP Content Determination in FM

**Figure 3:**
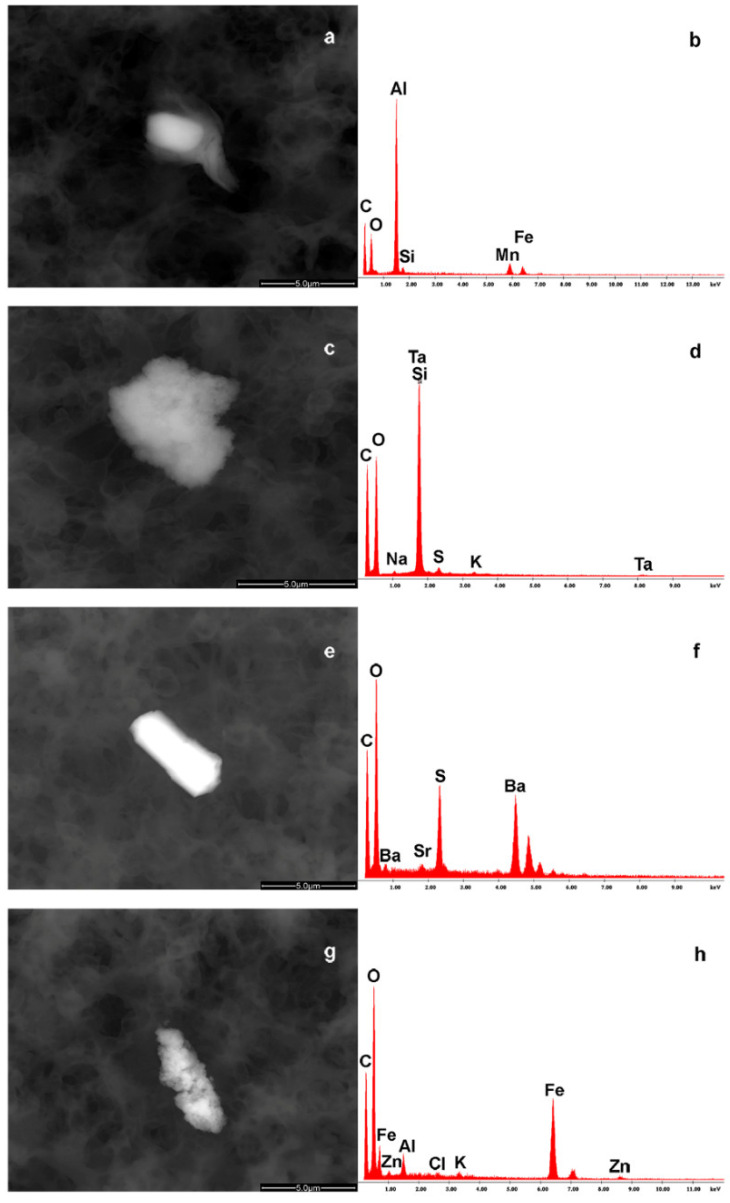
The images show some of the debris found in the FM with their EDS spectra. Some NP agglomerates were identified and characterized for the presence of aluminum (Al)–manganese (Mn)–iron (Fe)–silicon (Si) (**a**,**b**), tantalum (Ta)–Si–strontium (Sr) (**c**,**d**), sulfur (S)–barium (Ba)–Sr (**e**,**f**), or Fe–zinc (Zn)–Al–chlorine (Cl)–potassium (K) (**g**,**h**).

The elemental composition of the FM samples detected by means of EDS investigation is reported in Table 2. An EDS spectrum (42%) free from particulate pollution was observed in eight out of nineteen samples. In the 11 remaining cases (58%), we observed the presence of debris also forming agglomerates, namely organic/inorganic entities. It is possible to identify the presence of metallic debris, like aluminum–manganese–iron–silicon (a,b), tantalum–silicon–strontium (c,d), sulfur–barium–strontium (e,f), or iron–zinc–aluminum–chlorine–potassium (g,h). Also, in these cases, there is a common presence of carbon and oxygen in the milk substrate (Figure 3).

The chemical composition of these foreign bodies indicates a possible industrial origin during the process of production.

### 3.3. NP Content and Size: BM vs. FM

In Table 2, the difference in single elements between the BM and FM samples is reported. In particular, Ta, Mn, and Sr were not detectable in all BM samples analyzed. On the other hand, Rh, Pd, Au, Cu, Ni, and Cr were not identified in all FM samples analyzed. Finally, a significantly higher NP size (*p* < 0.05) was observed in the FM agglomerates than in the BM ones.

## 4. Discussion

Pollution is a growing worldwide hazard, especially regarding micro- and nanosized contamination, which accounts for the majority of unfortunate consequences [25,26]. In perinatal medicine, this contamination may be said to look like Janus’s face. On the one hand, the first stage of contaminant transmission starts in pregnancy, during which foreign bodies, especially nanosized ones, have been found to affect whole body organs [27]. On the other hand, the main source of micro- and NP transmission in the first months from birth is due to BM and FM feeding. The explanation lies in human lifestyle, exposure to organic and inorganic NPs, especially if they are composed of HM, through air, water, and food consumption [28]. Although the WHO has provided elemental safety ranges for HM, no general consensus has been reached regarding the side effects of HM contamination in milk by-products in infancy [29,30].

In the present study, we found an EDS spectrum: (i) clean in 37% of BM and 42% of FM samples, (ii) characterized by HM NP contamination in 63% of BM and 58% of FM samples, (iii) showing the presence of larger HM NP agglomerates in FM than BM fluids, and (iv) the presence of NP HM related to women living in industrial areas. To the best of our knowledge, this is the first report on EDS performance on BM and FM fluids.

The finding of a clean EDS spectrum in BM and FM fluids is not surprising and fits, at least in part, with previous observations [31]. The discrepancies can be explained in different (i) measurement techniques (EDS vs. mass spectroscopy); (ii) HM reference ranges varying from country to country [32]; and (iii) women’s lifestyle choices (i.e., tobacco smokers/non-smokers, passive smokers), dietary habits, and environmental factors (industrial/non-industrial regions) known to be responsible for several HM NP findings, such as Cr, Pb, and Cd [32,33,34]. Altogether, the absence in BM of other elements may be related to different lifestyle issues rather than to measurement techniques. Furthermore, the differences in composition in FM samples may be due to special products for infant characteristics, such as milk for preterm/term infants and/or special milk [32].

In this series, we found the presence of HM NPs in agglomerates both in BM and FM samples, with a significantly greater size in FM ones. The finding of HM NPs in agglomerates constitutes the first report in this setting. The explanation may lie in the advantages provided by FEGESEM-EDS technology.

The findings regarding the presence and the size differences in HM NP agglomerates between BM and FM fluids deserve further consideration. In particular, as to HM NPs, (i) once entered into the human body, they are carried through the systemic circulation to different organs and, in this case, to the breast, thus entering the milk; (ii) their primary targets are the proteins, highly concentrated in milk, thus constituting HM–NPs–milk–ligand complexes, namely agglomerates through a nano-biointeraction process [35], and the data is consistent with previous observations confirming the ability of HM NPs to evade the natural defenses of the organism and to interact directly with proteins, enzymes, DNA, etc. [36]; and (iii) there is the presence of agglomerates, varying between BM and FM both in elemental composition and size. The unusual chemical compounds found can be explained by women’s lifestyles involving HM NP exposure through inhalation, ingestion, or the intake of other substances that have escaped the observation criteria. This especially holds for the debris containing Ba, Ti, Pd, Au, Cu, Zn, and Ni, not uncommon air/water pollutants in humans living in urban and industrial/mining areas [34]. The debris of Figure 2 containing Au, Pd, Rh, Fe, and Ni induces one to think of a dental origin, namely it can be debris ingested during dental procedures on golden bridges using stainless-steel burs [30].

It is also conceivable that the presence of Fe may not be related to either diet or prenatal supplementation of Fe ions [37], since it is tightly linked to chromium forming an alloy like stainless-steel. The presence of elements such as Ba, Ti, Zn, Ta, Si, Al, and Sr points toward pollution rather than industrial preparation procedures. However, bearing in mind the strict rules for biological areas on animal farms, further multicenter investigations over a wider range of samples are required to clarify the hitherto unresolved questions.

The last point was characterized by a larger HM NP size in the FM samples. The explanation may lie in the higher total protein amount in FM vs. BM fluids. Other issues, such as temperature, pH, and contaminating ions, have been reported to interfere with the size and activity of HM NPs [31]. In addition, it has been shown that the greater the number of elements making up NPs, the lower the stability of the molecules leading to aggregate formation [38].

In this study, we have identified the following limitations: (i) the need for a larger number of BM and FM samples coming from the same area and company, respectively, in order to investigate potential NP changes in the same area and product; (ii) the absence of detailed information on women’s lifestyles and dietary regimes; (iii) the lack of quantitative NP measurement in different BM samples at different times; and (iv) the lack of short- and long-term infant follow-up. For these reasons, more in-depth studies conducted on a larger number of data are necessary.

With more data available, industrial products could be improved, and preventive measures could be indicated relating to lifestyle, nutrition, use of drugs, and controls to be carried out on both humans and animals.

## Figures and Tables

**Table 1:** Characteristics of the mother–newborn dyad recorded at admission. Data are reported as median ± SD.

Parameters	NP (n = 19)
Mother Characteristics	
Maternal age, (y)	34 ± 3
Weight, (Kg)	63 ± 3
Height, (cm)	166 ± 5
Exclusive breastfeeding, n/total	19/19
Newborn Characteristics	
GA, (wks)	37 ± 3
BW, (g)	2895 ± 188
Gender, (M/F)	9/10
Delivery mode	
Caesarean section, (n/total)	0/19
Vaginal, (n/total)	19/19
Apgar score > 7	
at 1 min, n/total	19/19
at 5 min, n/total	19/19

Abbreviations: NP, nanoparticles; y, years; Kg, kilograms; cm, centimeters; GA, gestational age; wks, weeks; BW, birthweight; g, grams; M, male; F, female.

**Table 2:** The chemical composition of elements detected (+) or not (−) in breast and formula milk samples.

Particle Composition	Breast Milk	Formula Milk
*Basic milk composition*		
C	+	+
O	+	+
Ca	+	+
K	+	+
Cl	+	+
P	+	+
Mg	+	−
Na	+	+
*Particle composition*		
Ba	+	+
S	+	+
Ti	+	+
Zn	+	+
Ta	−	+
Rh	+	−
Pd	+	−
Au	+	−
Cu	+	−
Ni	+	−
Fe	+	+
Cr	+	−
Si	+	+
Al	+	+
Mn	+	−
Sr	−	+

**Table 3:** Microparticle and NP agglomerates identified in breast and formula milk.

Breast Milk	Particle Size
BaS, COSBaCa, COTiCa, COSCaKClP, COZnTi,CRhPdAuOCuCaNiFeZn	1–2 μm
FeCO, COClSK	2–5 μm
COCaPSCl, CFeOCrCaSSi, CSiOCaS	3–6 μm
COCaClK, CFeOTiCaAlMg	4 μm
COCaS, COSiAlKCaSMgNa	2 μm
**Formula Milk**	
COCaPSK	0.5 μm
COCaPS, AlCOMnFeSi, CCaOPSK	3–5 μm
COCaSPK, CCaOSPKCl, COTiCaPSK	3–5 μm
COCaKPS, SiOCSTaKNa, CFeO	3–7 μm
COCaKSP, OCSBaSr	6 μm
COCaPSK, OCFeAlZnKCl	4–7 μm

Abbreviations: Ba, barium; S, sulfur; C, carbon; O, oxygen; Ca, calcium; Ti, titanium; K, potassium; Cl, chlorine; P, phosphorus; Zn, zinc; Rh, rhodium; Pd, palladium; Au, gold; Cu, copper; Ni, nickel; Fe, iron; Cr, chromium; Si, silicon; Al, aluminum; Mg, magnesium; Na, sodium; Mn, Manganese; Ta, tantalum; Sr, strontium.

## Data Availability

The original contributions presented in the study are included in the article, further inquiries can be directed to the corresponding author.
